# Longitudinal actigraphy study on sleep patterns under reduced social restrictions in Japanese university students

**DOI:** 10.1186/s40101-025-00397-4

**Published:** 2025-06-10

**Authors:** Yuna Enomoto, Hiroko Kubo

**Affiliations:** 1 Cooperative Major in Human Centered Engineering, Graduate School of Humanities and Sciences, Nara Women’s University, Kitauoyanishi-Machi, Nara, 630-8506 Japan; 2 Faculty of Engineering, Nara Women’s University, Kitauoyanishi-Machi, Nara, 630-8506 Japan

**Keywords:** Actigraphy, Sleep Patterns, Social restrictions, University Students, COVID-19, Circadian Rhythms, Total Sleep Time, Sleep Variability, Sleep Duration

## Abstract

**Background:**

Sleep deprivation and irregular sleep patterns can adversely affect physical and mental health. The COVID-19 pandemic presented a naturalistic opportunity to examine how reduced social time restrictions influence sleep behavior. This study aimed to investigate both group-level and individual-level changes in sleep patterns among Japanese university students before and during the pandemic and to explore how individual characteristics may contribute to these changes.

**Methods:**

Twenty-two female university students wore waist-worn actigraphy devices for approximately 16 weeks in both 2019 and 2020. Objective sleep data were collected alongside questionnaire assessments of chronotype, personality traits, and subjective sleep feeling.

**Results:**

In total, 4,432 valid days of actigraphy data were analyzed. Compared with the pre-pandemic year, sleep timing was delayed by approximately 20 min for bed-in time and 40 min for bed-out time in 2020. Time in bed (TIB) increased by about 20 min, while total sleep time (TST) remained largely unchanged. Sleep efficiency declined, but subjective sleep feeling remained stable. Individual-level analyses revealed substantial variability: 9 of 22 participants showed significant changes in TST, with both increases and decreases observed. Increased TIB was associated with later bed-out time, shorter baseline sleep duration, and lower neuroticism. A later bed-in time was associated with reduced TST.

**Conclusions:**

These findings suggest that while social time restrictions can influence sleep timing and duration, the effects vary considerably across individuals. Earlier bedtimes may be more effective than simply extending TIB in promoting adequate sleep. Furthermore, individual characteristics such as personality traits may play a role in sleep adaptation under changing social contexts. Given the diversity of responses observed, both group- and individual-level perspectives are essential for understanding sleep behavior in real-world settings.

## Background

Sleep deprivation has various negative effects on human health, including physiological and psychological aspects [1–8], and economic productivity [9]. According to the Organization for Economic Cooperation and Development, Japanese people sleep less than people in any other country. Additionally, 37.5% of men and 40.6% of women in Japan sleep < 6 h per day [10], highlighting a serious public health concern.

In evaluating sleep, both its quantity and consistency are important. Irregular sleep patterns—such as large daily variations in bedtime and duration—have been associated with adverse health outcomes [11, 12]. One factor contributing to such irregularities is social jetlag [13], which describes the misalignment between an individual’s biological rhythms and social schedules. While social jetlag often refers to differences between weekdays and weekends, it is conceptually linked to broader fluctuations in social time restrictions. Understanding how sleep patterns change in response to these fluctuations can offer insights into sleep stability and the factors that promote or disrupt it. However, most existing studies have focused on population-level averages, and relatively little attention has been paid to how individuals differentially adapt their sleep to changing social time pressure.

The coronavirus disease 2019 (COVID-19) pandemic provided a unique opportunity to investigate the relationship between social time restrictions and sleep behavior. Unlike experimental settings where sleep schedules are artificially manipulated, the COVID-19 pandemic provided a real-world scenario in which social restrictions were temporarily relaxed. This allows a naturalistic examination of how individuals adjust their sleep behaviors in response to reduced social time pressure.

The Japanese government’s policies, such as restrictions on nonessential outings, recommendations for remote work and study, and the suspension or reduced operation of settings that typically involve large gatherings (e.g., restaurants, event halls, and cultural facilities), temporarily altered social time restrictions. During this period, college students, who typically exhibit delayed sleep–wake patterns due to relatively flexible schedules [14, 15], experienced more schedule flexibility due to the shift to online learning, reduced part-time work, and cancelation of extracurricular activities. These unprecedented changes helped us examine how the relaxation of social restrictions influenced sleep behavior among university students, offering a context in which to explore how sleep schedules might adapt to changes in external social time pressure.

In addition to external social conditions, individual characteristics may also play a role in how people adapt their sleep behavior. Personality traits, for instance, have been associated with sleep-related outcomes in previous studies [16–18].

Therefore, this study aimed to investigate how reduced social time restrictions during the COVID-19 pandemic affected sleep behavior among university students, both at the group and individual levels, with a particular focus on variations in sleep timing and duration.

## Methods

### Participants

This study included first- and third-year university students recruited in April 2019 via announcements in classes and in-person invitations on campus. A total of 31 female students (16 first-year and 15 third-year students) were enrolled in the study. Participants received written and oral explanations, and written informed consent was obtained from all. In April 2020, all 31 students were invited to participate in a follow-up survey, and 25 agreed to participate. For the 2020 survey, participants received written explanations by mail, and written informed consent was again obtained. Of these 25 participants, 22 were included in the final analysis. Participants were excluded if their actigraphy data acquisition rate was below 50% in either 2019 or 2020. Additionally, one participant with sufficient data was excluded due to a markedly advanced sleep–wake pattern unrelated to the pandemic context.

All included participants were healthy female university students. None were engaged in shift work or had diagnosed medical conditions known to affect sleep.

This study was approved by the Ethics Committee of Nara Women’s University (approval numbers: 17–08 [approved on May 22, 2019] and 20–23 [approved on October 1, 2020]).

### Procedure

The study was conducted during the university’s regular spring semesters: from April to August in 2019, and from May to September in 2020. The 2020 semester and data collection began one month later than usual due to the COVID-19 pandemic.

Participants were instructed to wear actigraphy devices throughout the measurement periods while maintaining their usual routines. In 2019, participants visited the laboratory once a month so that researchers could download data from the devices, which required periodic retrieval due to memory limitations. In 2020, some participants exchanged devices by mail due to pandemic-related restrictions.

At the beginning of each year’s measurement period, participants completed questionnaires assessing demographic and lifestyle factors, sleep habits, and chronotype, as described in a later section. These questionnaires were completed in person in 2019 and returned by mail in 2020. Additionally, personality questionnaire was sent and returned by mail near the end of the 2020 measurement period due to logistical constraints.

To account for environmental context, meteorological data such as average monthly temperature, humidity, and total daylight hours were obtained from the Japan Meteorological Agency (Nara location) [19]. In 2019 (April–August), temperature ranged from 13.5 °C to 28.3 °C, humidity from 56 to 80%, and daylight hours from 104 to 262 h. In 2020 (May–September), temperature ranged from 19.9 °C to 29.7 °C, humidity from 65 to 82%, and daylight hours from 72 to 262 h.

### Measurements

#### Measurements of sleep and activity using actigraphy devices

Daily sleep and activity patterns were objectively assessed using waist-worn actigraphy devices (model FS-750, FS-760, or FS-770, ACOS Co., Ltd., Nagano, Japan; size approx. 75 × 33.5 × 10.5 mm3). These devices recorded the acceleration and directional data every 2 min. The participants were instructed to clip the device to the front of their waist and wear it as continuously as possible, except when bathing. Actigraphy data obtained using the devices were analyzed using Sleep Sign Act Ver. 2 (Kissei Comtec Corporation, Nagano, Japan), which employs a proprietary algorithm that identifies “sleep” and “wake” states based on acceleration and directional data. This algorithm has been validated with approximately 85% agreement with polysomnography data. Its accuracy is comparable to or greater than that of wrist-worn actigraphy devices commonly used in sleep studies [20]. In addition to daily step counts, 11 sleep variables were analyzed, including bed-in and bed-out times, sleep onset and wake times, time in bed (TIB), total sleep time (TST), sleep latency, sleep efficiency (TST/TIB ratio), number of wakes after sleep onset (WASO), total WASO time, and latency from waking up to getting out of bed (bed-out latency).

Sleep bed-in and bed-out times determined by the actigraphy software were cross-checked against the participants’ daily sleep diaries to ensure data accuracy. Minor adjustments were made based on the posture and acceleration data when necessary. Additionally, periods incorrectly classified as major sleep during the daytime or when the device was likely removed were conservatively excluded. All data processing was performed by the same researcher using a consistent approach across 2019 and 2020 to maintain consistency.

To explore potential seasonal influences in actigraphy-based variables, the measurement period in each year was subdivided into four (2019) or three (2020) time blocks based on calendar months and corresponding meteorological conditions.

#### Assessment of subjective sleep feeling

Subjective sleep feeling was assessed using the Oguri-Shirakawa-Azumi Sleep Inventory Middle-Aged version (OSA-MA) [21] immediately after waking up on 7–12 days in 2019 and 2020. The OSA-MA comprises 16 questions, each with four response options. Scores are calculated for five factors: Factor 1 (sleepiness on rising), Factor 2 (initiation and maintenance of sleep), Factor 3 (frequency of dreaming), Factor 4 (refreshing), and Factor 5 (sleep length). The OSA-MA has been standardized using data from 670 Japanese individuals. The OSA-MA has also been employed in international publications involving Japanese populations [22, 23]. Nevertheless, its applicability to populations outside of Japan should be interpreted with caution.

#### Assessment of participants’ characteristics

##### Basic information questionnaire

A self-administered questionnaire was used to collect information on demographic characteristics (e.g., age, living arrangement, height, and weight), academic schedules (e.g., commuting time, class times), and lifestyle factors (e.g., sleep habits, physical activity, and dietary patterns). Several lifestyle-related items were adapted from the Tokyo Metropolitan Institute for Neurosciences Life Habit Inventory (TMIN-LHI) [24], a widely used Japanese instrument. While these variables were not directly analyzed in the present study, the information helped contextualize participant characteristics and supported consistency checks with other data sources.

##### Chronotype

The Japanese version [25] of the Morningness–Eveningness Questionnaire (MEQ) [26] was administered at the start of each year’s survey to assess chronotype characteristics. The questionnaire consists of 19 questions with scores ranging from 16 to 86. Individuals are categorized into five types based on their scores: 16–30 points as “obviously evening type,” 31–41 as “slightly evening type,” 42–58 as “intermediate type,” 59–69 as “slightly morning type,” and 70–86 as “obviously morning type.” In this study, the first two groups were combined as “evening type,” and the latter two groups were combined as “morning type.”

##### Personality characteristics

The Maudsley Personality Inventory (MPI) [27] was administered near the end of the 2020 measurement period to assess personality traits. The MPI consists of two subscales—Extraversion and Neuroticism—each comprising 24 items, scored 0–48, with higher scores indicating greater trait expression. MPI was selected for its frequent appearance in sleep-related research and the availability of validated Japanese versions [28], making it a practical and contextually appropriate measure for this study.

### Statistical analysis

Statistical analyses were performed using IBM SPSS ver. 38 (IBM, Armonk, NY, USA). Comparisons of the means of activity and sleep variables between 2019 and 2020 were performed using the paired t-test, whereas comparisons of score categories, such as OSA-MA scores, were performed using the Wilcoxon signed-rank test. Within-individual comparisons were performed using an unpaired t-test for sleep variables and the Wilcoxon rank sum test for OSA-MA scores. The relationship between each variable and the change in the variables was analyzed using Spearman’s correlation test.

## Results

### Sample and participant characteristics

A total of 22 participants met the inclusion criteria and were included in the analysis. Table 1 summarizes participant characteristics. The average age at baseline was 19.0 years (range: 18–21). The average BMI was 20.4 (range: 18.1–23.8) in 2019 and 20.4 (range: 17.8–23.1) in 2020. Living arrangements were balanced, with 11 participants living with family and 11 living alone. The number of valid actigraphy days per participant ranged from 85 to 114 in 2019 and from 76 to 113 in 2020.

**Table 1:** Participant characteristics in 2019 and 2020

Variable	2019	2020
Age (years, at baseline)	19.0 ± 1.1	–
BMI	20.4 ± 1.4	20.4 ± 1.7
Living arrangement (family / alone)	10 / 12	11 / 11
Data acquisition days (mean ± SD, range)	105.5 ± 7.0 (85–114)	96.0 ± 10.2 (76–113)

Chronotype scores (MEQ) were generally stable across the study period, with no significant difference observed between 2019 (48.8 ± 10.9) and 2020 (47.9 ± 10.0). Based on the criteria described in the Methods, 13 participants (59%) were classified as intermediate type, 4 (18%) as morning type, and 5 (23%) as evening type in 2019; in 2020, 15 (68%) were intermediate, 2 (9%) morning, and 5 (23%) evening. This distribution is broadly consistent with previous findings in young adult populations in Japan [25].

Personality trait scores (MPI) were available only for 2020. The mean extraversion score was 19.7 ± 10.3 and the mean neuroticism score was 25.6 ± 11.6. Given that the midpoint of each scale is 24, participants tended to be slightly more introverted, with average levels of neuroticism.

The sample size of 22 participants in this study is comparable to that of previous actigraphy-based studies on sleep patterns. A previous study on COVID-19-related sleep changes included 20 participants [29], and another actigraphy-based study on adolescent sleep included 16 participants [30], indicating the appropriateness of our sample size.

### Changes in daily sleep and activity

The mean number of steps per day was 8,536 ± 1,953 in 2019, which significantly decreased to 5,831 ± 2,515 in 2020.

The mean bed-in time was 0:43 ± 0:55 a.m. in 2019 and 1:04 ± 1:05 a.m. in 2020, indicating a delay of approximately 20 min. The mean bed-out time was 7:50 ± 1:19 a.m. in 2019 and 8:31 ± 1:03 a.m. in 2020, showing a delay of approximately 40 min. Although time in bed (TIB) increased by 19 min, total sleep time (TST) changed by only about 6 min.

Sleep latency was prolonged, sleep efficiency declined, the number of wake episodes after sleep onset (WASO) increased, and bed-out latency was extended.

In addition, seasonal variation was observed in the pre-pandemic year, with longer TIB and earlier bedtimes in April 2019. These patterns are consistent with well-known seasonal effects on sleep behavior. However, in 2020, TIB and bedtime remained extended and delayed across all seasons compared to their 2019 counterparts, suggesting that the impact of reduced social restrictions during the pandemic may have exceeded typical seasonal influences.

Table 2 summarizes the activity and sleep variables for each year.

**Table 2:** Activity and sleep variables and comparison of values between 2019 and 2020

	2019	2020	*t* value	Effect size (Cohen’s *d*)	*p* value	
	Mean SD	Mean SD				
Steps (steps/day)	8536 1953	5831 2515	6.058	1.292	0.000	*
Bed-in time (time)	0:43 0:55	1:04 1:08	− 1.880	− 0.401	0.074	n.s
Asleep time (time)	1:06 0:59	1:35 1:11	− 2.479	− 0.528	0.022	*
Awakening time (time)	7:39 1:20	8:19 1:03	− 3.612	− 0.770	0.002	*
Bed-out time (time)	7:50 1:19	8:31 1:03	− 3.780	− 0.806	0.001	*
TST (h:mm)	5:10 0:43	5:04 0:56	1.127	0.240	0.272	n.s
TIB (h:mm)	7:05 0:45	7:26 0:47	− 2.611	− 0.557	0.016	*
SL (h:mm)	0:23 0:14	0:31 0:17	− 3.531	− 0.753	0.002	*
SE (%)	73.3 9.1	68.6 12.1	3.981	0.849	0.001	*
Number of WASO (times)	11.3 2.9	12.3 3.2	− 2.039	− 0.435	0.054	n.s
Total time of WASO (h:mm)	1:22 0:31	1:39 0:47	− 3.003	− 0.640	0.007	*
Bed-out latency (h:mm)	0:10 0:04	0:11 0:05	− 2.450	− 0.522	0.023	*

*n.s. *Not significant, *TST *Total sleep time, *TIB *Time in bed, *SL *Sleep latency, *SE *Sleep efficiency, *WASO *Wake after sleep onset

^*^p < 0.05

As previously mentioned, no statistically significant difference was found in mean TST between 2019 and 2020. To further investigate this, we examined individual-level variation using each participant’s data collected over 176–224 days. Among the 22 participants, 13 showed no significant change in TST, while 5 exhibited a significant decrease and 4 a significant increase. These findings underscore notable individual differences in sleep responses, suggesting that group-level statistics alone may obscure meaningful personal patterns. Therefore, we further examined the factors associated with changes in TIB and TST.

### Factors associated with changes in sleep length

To examine the factors associated with changes in sleep length, we calculated the changes in TIB and TST between 2019 and 2020 (ΔTIB and ΔTST, respectively) for each participant and analyzed their correlations with the following variables: (1) TST in 2019, to examine its relationship with TST changes in 2020 and explore the possible role of prior sleep debt; (2) Changes in daytime steps (Δsteps), to assess their association with changes in sleep demand; (3) Changes in bed-in time (Δbed-in time), to investigate its relationship with changes in sleep length; (4) Changes in bed-out time (Δbed-out time), to explore how shifts in wake time may relate to sleep patterns; (5) Neuroticism, to examine whether emotional instability is associated with changes in sleep behavior; and (6) Extraversion, to assess whether social and behavioral tendencies influence sleep adjustments under altered social conditions. Table 3 presents the correlations between ΔTIB, ΔTST, and these variables.

**Table 3:** Correlations of ΔTIB and ΔTST with each variable

	ΔTST		ΔTIB	
	ρ (*p* value)		ρ (*p* value)	
TST in 2019	− 0.006 (0.978)	n.s	− 0.580 (0.005)	*
ΔSteps	− 0.171 (0.446)	n.s	− 0.280 (0.208)	n.s
Δbed-in time	− 0.589 (0.004)	*	− 0.335 (0.128)	n.s
Δbed-out time	− 0.129 (0.566)	n.s	0.520 (0.013)	*
Neuroticism	− 0.143 (0.525)	n.s	− 0.401 (0.064)	n.s
Extraversion	− 0.364(0.096)	n.s	− 0.181 (0.419)	n.s

*n.s. *Not significant, *TST *Total sleep time, *TIB *Time in bed

^*^p < 0.05

Changes were calculated as 2020 minus 2019 values; thus, smaller values indicate shorter TIB or TST, fewer steps, or an earlier sleep schedule than the previous year. Higher neuroticism scores reflect greater emotional instability, and higher extraversion scores reflect greater extraversion

TST in 2019 was significantly correlated with ΔTIB, suggesting that individuals with shorter sleep duration before the pandemic were more likely to extend their time in bed. However, TST in 2019 was not significantly associated with ΔTST. Changes in daytime steps were not significantly correlated with either ΔTIB or ΔTST.

**Fig. 1:**
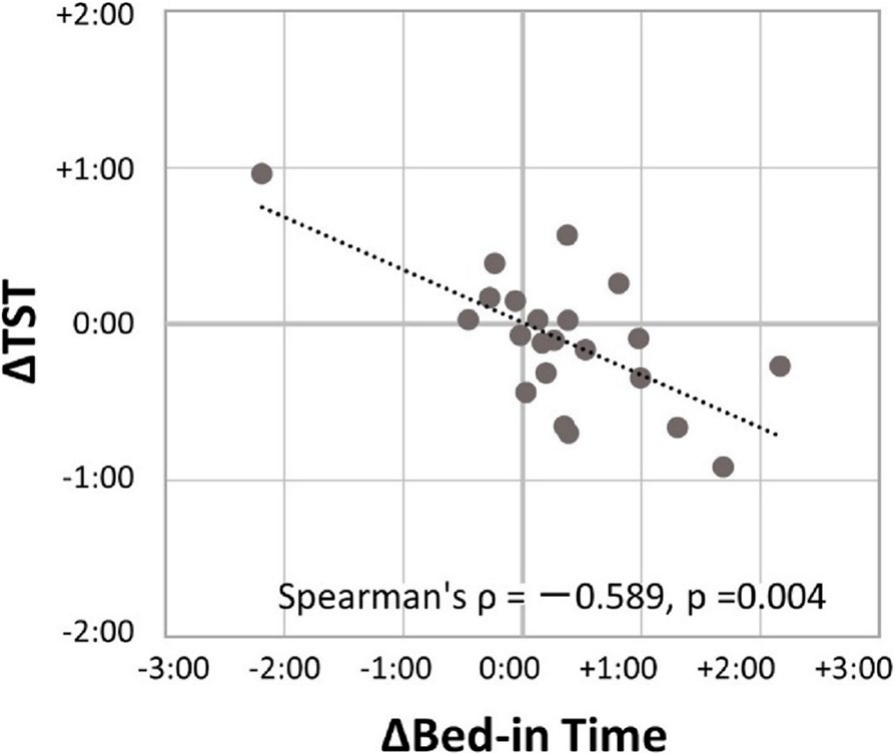
Relationship between changes in bed-in time and total sleep time (TST). TST decreased in participants whose bed-in time was delayed. Spearman’s *ρ* = − 0.589, *p* = 0.004; *n* = 22

As shown in Fig. 1, Δbed-in time showed a significant negative correlation with ΔTST (*ρ* = − 0.589, *p* = 0.004), indicating that those who went to bed later tended to sleep shorter.

**Fig. 2:**
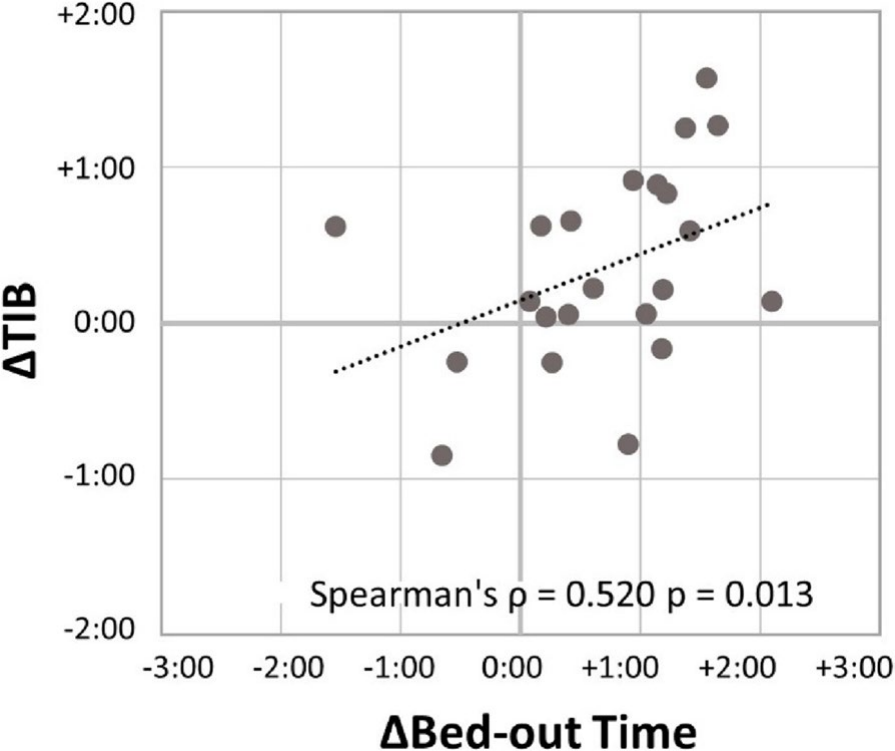
Relationship between changes in bed-out time and time in bed (TIB). TIB increased in participants whose bed-out time was delayed. Spearman’s *ρ* = 0.520, *p* = 0.013; *n* = 22

Δbed-out time showed a significant positive correlation with ΔTIB (*ρ* = 0.520, *p* = 0.013), suggesting that waking up later was associated with a longer time spent in bed (Fig. 2).

Neuroticism was not significantly correlated with either ΔTST or ΔTIB, although a weak negative correlation was observed for the latter (*ρ* = − 0.401, *p* = 0.064). Extraversion was also not significantly correlated with changes in TIB or TST.

### Assessment of objective sleep feeling

**Fig. 3:**
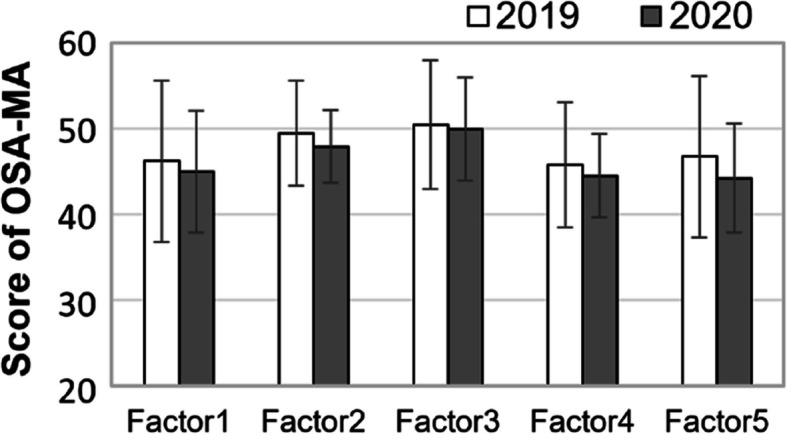
Mean OSA-MA scores for each factor in 2019 and 2020. Higher scores indicate better subjective sleep feeling in the morning. The OSA-MA evaluates five factors: (1) sleepiness on rising, (2) initiation and maintenance of sleep, (3) frequency of dreaming, (4) refreshing, and (5) sleep length. *n* = 22 for each year

Figure 3 shows the mean OSA-MA scores for each factor in 2019 and 2020. Although the average scores for all five factors were slightly lower in 2020, none of the changes were statistically significant. At the individual level, 10 out of 22 participants showed noticeable differences in at least one factor. The most frequently observed change was in the “sleep length” factor, with 3 participants reporting improvement and 2 reporting a decline in subjective sleep feeling.

## Discussion

### Changes during the pandemic

Several changes in sleep behavior were observed between the pre-pandemic (2019) and pandemic (2020) periods, when social time restrictions were substantially reduced. Previous studies have widely reported delayed sleep timing [30–36] and increased time in bed [29, 30, 32–36] during the pandemic period, and our results showed a similar trend.

The primary changes observed included a delay in sleep timing, with bed-in and bed-out times shifting approximately 20 and 40 min later, respectively. Although time in bed (TIB) increased by about 20 min, total sleep time (TST) was largely unchanged. Additionally, sleep quality indicators declined, with increased wake after sleep onset (WASO) and reduced sleep efficiency. The more pronounced delay in bed-out time compared to bed-in time suggests that participants adapted their wake-up schedules more flexibly than their bedtimes.

While many previous studies have reported increased sleep duration during the pandemic, they often relied on self-reported measures and primarily assessed TIB rather than TST. TIB is partially under voluntary control and can be extended by staying in bed longer, whereas TST reflects actual time spent asleep, determined by physiological sleep processes. In our study, although TIB increased, TST was largely unchanged. This discrepancy suggests that TIB and TST capture distinct facets of sleep and may warrant separate consideration when evaluating the impact of social factors.

Despite the decline in sleep efficiency, subjective sleep feeling, as assessed by standardized questionnaire scores, did not significantly change. Some participants even showed improved scores, which may suggest that the reduction in social time restrictions had a positive effect on sleep perception for certain individuals. The OSA-MA, while widely used in Japanese populations, is less common internationally and should therefore be interpreted with some caution.

Nevertheless, a nationwide Japanese survey reported a decrease in complaints of nonrestorative sleep during the pandemic, despite global concerns over deteriorating sleep quality [37]. This pattern shows some similarity to our findings, in which subjective sleep feeling remained stable—or modestly improved—for some participants, even as objective indicators declined. These results may indicate that, particularly in Japanese populations, reduced social restrictions contribute to improvements in perceived sleep quality, regardless of physiological changes.

### Associations with changes in sleep length

A key strength of this study was its longitudinal within-subject design, which allowed us to assess not only group-level differences but also individual variations in sleep changes. Although the mean TST did not differ significantly between 2019 and 2020, approximately 9 out of 22 participants showed statistically significant changes in TST—with 5 participants experiencing a significant decrease and 4 experiencing a significant increase. This observation points to the possibility that group-level statistics may not fully capture individual variation.

We examined potential factors influencing these changes, focusing on TIB and TST. The increase in TIB was associated with shorter sleep duration in 2019, lower neuroticism, and a delay in bed-out time. This aligns with findings by Korman et al. [35], who suggested that individuals with prior sleep debt may have compensated by staying in bed longer during the pandemic. The increase in TIB was primarily associated with a delay in bed-out time rather than bed-in time, suggesting that the extension of time in bed resulted mainly from later wake-up times.

Previous research has explored associations between neuroticism and various aspects of sleep, such as longer sleep duration [38] and poorer subjective sleep quality [39], though findings have been inconsistent. In our study, neuroticism was associated with changes in time in bed (TIB) but not total sleep time (TST). This pattern floats the possibility that personality traits such as neuroticism influence how individuals adjust their sleep schedules, rather than directly determining physiological sleep duration. Given that social time restrictions vary in daily life, further research may help clarify how individual characteristics shape sleep variability under different social conditions.

A later bed-in time was associated with shorter TST, suggesting that bedtime adjustments may play a crucial role in determining sleep duration—consistent with studies showing that delayed bedtimes are linked to reduced sleep duration [40]. Some research has also suggested that a later bedtime may negatively impact health independent of sleep length [41, 42]. These findings are in line with previous research suggesting that earlier bedtimes may be more beneficial than simply extending time in bed.

### Limitations and strengths

This study has several limitations. First, our sample was limited to female university students residing in regional areas of Japan. While this group experienced reduced social time restrictions during the pandemic—such as the shift to online classes and suspension of extracurricular activities—their circumstances may differ from those of students in urban areas [36]. This limits the generalizability of the findings across populations.

Second, while all participants experienced pandemic-related changes such as remote classes and reduced social obligations, the extent of those changes likely varied. For instance, some students may have continued in-person research activities or part-time jobs depending on their individual circumstances. These variations were not fully captured in this study. A recent study among Japanese workers found that differences in work-related time restrictions (e.g., full-time versus part-time employment) were associated with sleep debt and well-being during the pandemic [43]. Similarly, subtle differences in students’ daily schedules may have influenced how they adapted their sleep, suggesting a need for more detailed assessment of social time restrictions in future research.

Third, environmental factors such as light exposure, which are known to influence circadian rhythms, were not directly assessed. While some ambient conditions—including light, temperature, and humidity—were recorded in the home environment, these data were not analyzed due to limited information on participants’ actual exposure. This limits our ability to fully account for environmental influences on sleep and circadian outcomes.

Finally, personality traits were assessed only in 2020, during the pandemic, and may have been influenced by the unusual social and psychological context of that period. Furthermore, the associations observed between individual characteristics and sleep behavior may not necessarily generalize to more typical fluctuations in social time restrictions. These findings should therefore be interpreted with caution, and future studies should examine whether similar patterns emerge under more routine social conditions.

Despite these limitations, this study had several strengths. Unlike many pandemic-related sleep studies that relied on self-reported or retrospective data, our study objectively tracked sleep–wake patterns across two consecutive years using actigraphy. The within-subject longitudinal design allowed us to explore not only overall trends but also individual variation in sleep adaptation, which may offer a more nuanced perspective on how reduced social time restrictions relate to sleep patterns.

## Conclusion

This longitudinal study examined how reduced social time restrictions during the COVID-19 pandemic affected sleep patterns in Japanese female university students. At the group level, we observed delayed sleep timing, extended time in bed, and decreased sleep efficiency, whereas total sleep time and subjective sleep feeling remained relatively stable.

While group-level differences in total sleep time were not significant, individual-level analyses revealed considerable variation: 9 out of 22 participants showed significant changes in sleep duration, with both increases and decreases observed. These results illustrate the importance of examining not only average trends but also inter-individual differences when investigating sleep adaptation under changing social contexts.

We also observed that certain personality traits, such as lower neuroticism, might be linked to behavioral adjustments in sleep schedules. While preliminary in nature, these findings may inform future research on individual variability in sleep responses.

## Data Availability

The datasets generated and analyzed during the current study are not publicly available due to privacy considerations but are available from the corresponding author upon reasonable request.

## Abbreviations

OSA-MA: Oguri-Shirakawa-Azumi Sleep Inventory Middle-Aged
TIB: Time in bed
TST: Total sleep time
WASO: Wakes after sleep onset
